# Cysteine-Mediated Gene Expression and Characterization of the CmbR Regulon in *Streptococcus pneumoniae*

**DOI:** 10.3389/fmicb.2016.01929

**Published:** 2016-12-01

**Authors:** Muhammad Afzal, Irfan Manzoor, Oscar P. Kuipers, Sulman Shafeeq

**Affiliations:** ^1^Department of Molecular Genetics, Groningen Biomolecular Sciences and Biotechnology Institute, University of GroningenGroningen, Netherlands; ^2^Department of Bioinformatics and Biotechnology, Government College UniversityFaisalabad, Pakistan; ^3^Department of Microbiology, Tumor and Cell Biology, Karolinska InstitutetStockholm, Sweden

**Keywords:** Cysteine, CmbR, pneumococcus, MetE, MetA

## Abstract

In this study, we investigated the transcriptomic response of *Streptococcus pneumoniae* D39 to cysteine. Transcriptome comparison of the D39 wild-type grown at a restricted concentration of cysteine (0.03 mM) to one grown at a high concentration of cysteine (50 mM) in chemically-defined medium (CDM) revealed elevated expression of various genes/operons, i.e., *spd-0150*, *metQ*, *spd-0431*, *metEF*, *gshT*, *spd-0618*, *fhs, tcyB*, *metB*-*csd*, *metA*, *spd-1898*, *yvdE*, and *cysK*, likely to be involved in the transport and utilization of cysteine and/or methionine. Microarray-based data were further confirmed by quantitative RT-PCR. Promoter *lacZ*-fusion studies and quantitative RT-PCR data showed that the transcriptional regulator CmbR acts as a transcriptional repressor of *spd-0150*, *metEF*, *gshT*, *spd-0618*, *tcyB*, *metA*, and *yvdE*, putatively involved in cysteine uptake and utilization. The operator site of CmbR in the promoter regions of CmbR-regulated genes is predicted and confirmed by mutating or deleting CmbR operator sites from the promoter regions of these genes.

## Introduction

The major human pathogen *Streptococcus pneumoniae* colonizes the human nasopharynx and is the causal agent of many diseases, including pneumonia, sepsis, meningitis, and others. Pneumococcal nitrogen metabolism and regulation have been studied extensively as the appropriate acquisition and metabolism of nutrients are important for its lifestyle (Hendriksen et al., 2008). Sulfur is an integral part of many essential components of the cell, such as cysteine, methionine, thiamine, biotin, lipoic acid, coenzyme A, etc. Among these compounds, cysteine plays a key role, as it is the most important sulfur-containing compound-forming metabolite and its *de novo* synthesis signifies the central pathway of sulfur acquisition in microorganisms and plants (Sperandio et al., 2005). Many important proteins (such as cytochromes and aconitase) also have cysteine as an essential amino acid in their catalytic domains. Moreover, cysteine (and the dimer cysteine) helps in protein folding, assembly and stability, being involved in the formation of disulfide bounds. Cysteine-derived proteins (such as thioredoxin and glutathione) help in countering oxidative stress (Sperandio et al., 2005). Methionine is another sulfur-containing amino acid, regulating the initiation of translation and is vital to several methyl-transferase reactions (Sperandio et al., 2005). Microorganisms can synthesize methionine by converting homoserine to homocysteine through addition of a sulfur group from either cysteine (requiring MetABC), sulfide (requiring MetA and CysD) or by using the SAM (*S*-adenosylmethionine) recycling pathway (MetK, Pfs, and LuxS) (Kovaleva and Gelfand, 2007). Homocysteine is then methylated by methionine synthase (MetE) in conjunction with a methylenetetrahydrofolate reductase (MetF), with the methyl group supplied by 5-methyltetrahydrofolate, to form methionine (Kovaleva and Gelfand, 2007).

Cysteine and methionine concentrations might be regulating bacterial growth in different conditions, such as pathogenic events or fermentation processes, as these amino acids have essential roles in metabolism (Schell, 1993). In *Brucella melitensis* (Lestrate et al., 2000), *Haemophilus parasuis* (Hill et al., 2003) and, *Salmonella enterica* (Ejim et al., 2004), sulfur-containing amino acid biosynthesis genes have been characterized as virulence factors. The *cysDNC* operon involved in the sulfate activation pathway forms a stress-induced operon in *Mycobacterium tuberculosis* (Pinto et al., 2004), whereas several thiol- and cysteine metabolism genes comprise the *sigH* regulon necessary for optimal existence of the bacterium in macrophages (Manganelli et al., 2002). Furthermore, cysteine metabolism also controls the regulation of toxin formation in *Bordetella pertussis* (Bogdan et al., 2001). Similarly, cysteine regulates a signaling molecule derivative of sulfur metabolism, autoinducer 2, which is conserved in both Gram-positive and -negative bacteria and is involved in interspecies communication and regulation of virulence factors (Sperandio et al., 1999; Marouni and Sela, 2003).

Our current study elucidates the effect of cysteine on the global gene expression of *S. pneumoniae* and characterizes the role of the transcriptional regulator CmbR in regulation of *spd-0150*, *metEF*, *gshT*, *spd-0618*, *tcyB*, *metA*, and *yvdE*. The transcriptional regulator CmbR acts as a transcriptional repressor for a number of genes/operons involved in cysteine uptake and utilization. The putative operator site (5′-GYGATAAAAAWWAYTTATMAC-3′ where Y = T/C, W = A/T and M = A/C) of CmbR in the promoter regions of *spd-0150*, *metEF*, *gshT*, *spd-0618*, *tcyB*, *metA*, and *yvdE* is predicted and confirmed by promoter mutational/deletion experiments. Moreover, this site is found highly conserved in other pneumococcal strains and streptococci.

## Materials and Methods

### Bacterial Strains and Growth Conditions

Bacterial strains and plasmids used in this study are listed in **Table 1**. *S. pneumonia*e D39 was grown as described previously (Kloosterman et al., 2006a; Afzal et al., 2014). For β-galactosidase assays, derivatives of *S. pneumoniae* D39 were grown in a chemically defined medium (CDM) (Kloosterman and Kuipers, 2011) supplemented either with 0.03 or 50 mM cysteine. CDM was prepared without cysteine. For selection on antibiotics, the medium was supplemented with the following concentrations of antibiotics: tetracycline: 2.5 μg/ml for *S. pneumoniae*; ampicillin: 100 μg/ml for *Escherichia coli* and erythromycin: 0.25 μg/ml for *S. pneumoniae* and 120 μg/ml for *E. coli*. All bacterial strains used in this study were stored in 10% (v/v) glycerol at -80°C. For PCR amplification, chromosomal DNA of *S. pneumoniae* D39 (Lanie et al., 2007) was used as a template. Primers used in this study are based on the sequence of the *S. pneumoniae* D39 genome and are listed in **Table 2**.

**Table 1:** List of strains and plasmids used in this study.

Strain/plasmid	Description	Source
***S. pneumoniae***		
D39	Serotype 2 strain. *2*	Laboratory of P. Hermans.
MA1000	D39 Δ*cmbR*	This study
MA1101	D39 Δ*bgaA*:: P*spd-0150-lacZ*; Tet^R^	Afzal et al., 2016
MA1104	D39 Δ*bgaA*:: P*metE-lacZ*; Tet^R^	Afzal et al., 2016
MA1105	D39 Δ*bgaA*:: P*gshT-lacZ*; Tet^R^	Afzal et al., 2016
MA1106	D39 Δ*bgaA*:: P*spd-0618-lacZ*; Tet^R^	Afzal et al., 2016
MA1109	D39 Δ*bgaA*:: P*tcyB-lacZ*; Tet^R^	Afzal et al., 2016
MA1110	D39 Δ*bgaA*:: P*metA-lacZ*; Tet^R^	Afzal et al., 2016
MA1001	D39 Δ*bgaA*:: P*yvdE-lacZ*; Tet^R^	This study
MA1002	MA1000 Δ*bgaA*:: P*spd-0150-lacZ*; Tet^R^	This study
MA1114	MA1000 Δ*bgaA*:: P*metE-lacZ*; Tet^R^	This study
MA1003	MA1000 Δ*bgaA*:: P*gshT-lacZ*; Tet^R^	This study
MA1004	MA1000 Δ*bgaA*:: P*spd-0618-lacZ*; Tet^R^	This study
MA1005	MA1000 Δ*bgaA*:: P*tcyB-lacZ*; Tet^R^	This study
MA1006	MA1000 Δ*bgaA*:: P*metA-lacZ*; Tet^R^	This study
MA1007	MA1000 Δ*bgaA*:: P*yvdE-lacZ*; Tet^R^	This study
MA1008	D39 Δ*bgaA*:: P*spd-0150-M-lacZ*; Tet^R^	This study
MA1009	D39 Δ*bgaA*:: P*spd-metE-M-lacZ*; Tet^R^	This study
MA1010	D39 Δ*bgaA*:: P*spd-0618R1-M-lacZ*; Tet^R^	This study
MA1011	D39 Δ*bgaA*:: P*spd-0618R2-M-lacZ*; Tet^R^	This study
MA1012	D39 Δ*bgaA*:: P*metA-TER-lacZ*; Tet^R^	This study
***E. coli***		
EC1000	Km^R^; MC1000 derivative carrying a single copy of the pWV1 *repA* gene in *glgB*	Laboratory collection
**Plasmids**		
pPP2	Amp^R^ Tet^R^; promoter-less *lacZ*. For replacement of *bgaA* with promoter *lacZ* fusion. Derivative of pPP1	Halfmann et al., 2007
pORI280	Erm^R^; *ori^+^ repA^-^;* deletion derivative of pWV01; constitutive *lacZ* expression from P32 promoter	Leenhouts et al., 1998
pMA1000	pORI280 carrying *cmbR* deletion	This study
pMA1101	pPP2 P*spd-0150-lacZ*	Afzal et al., 2016
pMA1104	pPP2 P*metE-lacZ*	Afzal et al., 2016
pMA1105	pPP2 P*gshT-lacZ*	Afzal et al., 2016
pMA1106	pPP2 P*spd-0618-lacZ*	Afzal et al., 2016
pMA1109	pPP2 P*tcyB-lacZ*	Afzal et al., 2016
pMA1110	pPP2 P*metA-lacZ*	Afzal et al., 2016
pMA1001	pPP2 P*yvdE-lacZ*	This study
pMA1002	pPP2 P*spd-0150-M-lacZ*	This study
pMA1003	pPP2 P*metE-M-lacZ*	This study
pMA1004	pPP2 P*spd-0618R1-M-lacZ*	This study
pMA1005	pPP2 P*spd-0618R2-M-lacZ*	This study
pMA1006	pPP2 P*metA-TER-lacZ*	This study

**Table 2:** List of primers used in this study.

Name	Nucleotide Sequence (5′-3′)	Restriction site
spd-0150-M-F	CATGGAATTCGGTCTTTTAAATTACCCGCGAAAAAAACTTATCA	*EcoRI*
spd-0150-R	CATGGGATCCGGCAGCAAGAGATGAGTAT	*BamHI*
metE-M-F	CATGGAATTCATCAGTTATAGTCTTTTCTAATAACAAGCCATAGTCACTTGCAAGAATTACTAGCAACGC	*EcoRI*
metE-R	CATGGGATCCGTTGACATGATGTGTCCTCC	*BamHI*
spd-0618-M-R1-F	CATGGAATTCATGTCTATGGCCAAAAATCCTGCGAAC	*EcoRI*
spd-0618-M-R2-F	CATGGAATTCATGTCTATGGTAAAAAATCCTTATAACGGCAGCGAAAAATAGAGCGTAT	*EcoRI*
spd-0618-R	CATGGGATCCGTTCAACAATGGACCAATCC	*BamHI*
metA-CmbR-TER	CATGGAATTCGTTAGAGAAAAACTATAATTGAA	*EcoRI*
metA-CmbR-TER	CATGGAATTCGTTAGAGAAAAACTATAATTGAAAATTGTGTCT	*EcoRI*
metA-R	CATGGGATCCGCACGTTGATCATCCATGAC	*BamHI*
yvdE-F	CATGGAATTCGTCATTGAACGTGGTAACC	*EcoRI*
yvdE-R	CATGGGATCCCATAGATTTGCAGCAACTCC	*BamHI*
CmbR-1	TGCTCTAGACATTTATGCTAGTGGAGG	*XbaI*
CmbR-2	CCACTATTGGCAATAGCC	*–*
CmbR-3	CTATTGCCAATAGTGGCTCTTCGTAGAAGTCATGC	*–*
CmbR-4	GAAGATCTCCGTCAGATAGCTATCTGCC	*BglII*
**RT-PCR primers**		
gyrA-F	CGAGGCACGTATGAGCAAGA	–
gyrA-R	GACCAAGGGTTCCCGTTCAT	–
spd-0150-1	GCGGCTTGCTCAGGGGG	–
spd-0150-2	CCAGCAAAGACACCTGACC	–
metQ-1	GCTACAGTCGCAGGTTTGGC	–
metQ-2	CTTCGCCATCAGCAGTTGC	–
spd-0431-1	GGTCTGGTTGATGGTGCGG	–
spd-0431-2	CCAGTAATCCACCGTCTG	–
metE-1	GGCATCACTGAAATCCC	–
metE-2	GGTAACCACGTCCCAAAGCG	–
metF-1	CCGTCACTCTCATTTGAAG	–
metF-2	GGCAAGTGGGCAATGGTCG	–
gshT-1	CGTGCCACCATTTGACTACG	–
gshT-2	GCAGCCTGATAGTGACC	–
spd-0618-1	CGTTAGTATCATCCGAC	–
spd-0618-2	GATTCTGCCATATAGGAG	–
fhs-1	CAGATATTGAAATCGCACAG	–
fhs-2	GCCTTGTACTTTCCGTAC	–
Csd-1	CGTTTAGGGCACCATACC	–
Csd-2	GTCTTCACTGGCATAGG	–
metB-1	GTCAGATGAAGCGACAGG	–
metB-2	GCCAAGACTTCCTCAGCC	–
metA-1	GGCTAATACACCCCTACA	–
metA-2	CCTCAAATGGTAAATGCTC	–
spd-1898-1	GGAACATCTGGTCGTTC	–
spd-1898-2	CTATCATAACGCTTACC	–
yvdE-1	GGCTAGAACGGTTGTAGG	–
yvdE-2	CTGACTCATCACCAACAGG	–
cysK-1	CATCGTGCCAGAAGGTGC	–
cysK-2	CCTTTAGCAGCACCTACC	–

### Construction of a *cmbR* Mutant

A markerless *cmbR* mutant (MA1000) was constructed in *S. pneumoniae* D39 using pORI280, as described before (Kloosterman et al., 2006a). Primer pairs cmbR-1/cmbR-2 and cmbR-3/cmbR-4 were used to generate PCR fragments of the left and right flanking regions of *cmbR*. The integrity of the *cmbR* mutant (MA1000) was further confirmed by PCR and DNA sequencing.

### Construction of Promoter *lacZ*-Fusions and β-Galactosidase Assays

Chromosomal transcriptional *lacZ*-fusions to the *spd-0150*, *metE*, *gshT*, *spd-0618*, *tcyB*, *metA*, and *yvdE* promoters were constructed in our previous study (Afzal et al., 2016). These constructs were further introduced into the D39 Δ*cmbR* (MA1000) strain resulting in strains MA1002-06, respectively. Transcriptional *lacZ*-fusion to the *yvdE* promoter was constructed in pPP2 (Halfmann et al., 2007) with primer pairs mentioned in **Table 2** resulting in pMA1001. This construct was further introduced into the D39 wild-type and the D39 Δ*cmbR* (MA1000) strains resulting in strains MA1001 and MA1007, respectively. The following sub-clones of P*spd-0150*, P*metE*, P*spd-0618*, and P*metA* were made in pPP2 (Halfmann et al., 2007) using the primer pairs mentioned in **Table 2**: P*spd-0150-M* (mutation in the *cmbR* site), P*metE-M* (mutation in the *cmbR* site), P*spd-0618R1-M* (mutation in the *cmbR* site 1), P*spd-0618R2-M* (mutation in the *cmbR* site 2), and P*metA*-*TER* (termination of the *cmbR* site), resulting in plasmids pMA1002-06, respectively. These constructs were introduced into the *S. pneumoniae* D39 wild-type, resulting in strains MA1008-12, respectively. All plasmid constructs were checked for the presence of the insert by PCR and DNA sequencing.

β-galactosidase assays were performed as described before (Israelsen et al., 1995; Halfmann et al., 2007) using cells that were harvested in the mid-exponential growth phase, and grown in CDM supplemented either with 0.03 or 50 mM cysteine.

### Microarray Analysis

Microarray analysis was performed as described before (Afzal et al., 2015a; Shafeeq et al., 2015). For DNA microarray analysis of *S. pneumoniae* in the presence of cysteine, the transcriptomes of *S. pneumoniae* D39 wild-type, grown in replicates in CDM with 0.03 mM cysteine, was compared to that grown in CDM with 50 mM cysteine and harvested at respective mid-exponential growth phases. For the identification of differentially expressed genes, a Bayesian *p*-value of <0.001 and a fold-change cut-off >1.5 was applied. RNA isolation was performed as described before (Afzal et al., 2015a). All other procedures regarding the DNA microarray experiments and data analysis were performed as previously described (Shafeeq et al., 2011a,b; Afzal et al., 2015b). Microarray data have been submitted to GEO under the accession number GSE89458.

### Reverse Transcription (RT)-PCR and Purification for Quantitative RT-PCR

For quantitative RT-PCR, *S. pneumoniae* D39 wild-type and D39 Δ*cmbR* were grown in replicates in CDM supplemented with either 0.03 mM or 50 mM cysteine. RNA isolation was done as described before (Afzal et al., 2015a). First, strand cDNA synthesis was performed on RNA (Shafeeq et al., 2011b). cDNA (2 μl) was amplified in a 20 μl reaction volume that contained 3 pmol of each primer (**Table 2**) and the reactions were performed in three technical replicates on two biological replicates of RNA (Shafeeq et al., 2011b). The transcription level of specific genes was normalized to *gyrA* transcription, amplified in parallel with gyrA-F and gyrA-R primers. The results were interpreted using the comparative CT method (Schmittgen and Livak, 2008). Differences in expression of twofold or greater relative to control were considered as significant.

## Results

### Cysteine-Dependent Gene Regulation in *S. pneumoniae* D39

Cysteine is one of the most important amino acids for bacteria. It is also present in human blood plasma at concentration of 0.03 mM (Lopez, 2013). To study the impact of cysteine on the transcriptome of *S. pneumoniae* D39 wild-type, we performed microarray comparison of *S. pneumoniae* D39 grown in CDM with 0.03–50 mM cysteine. 0.03 mM concentration was chosen, as this is the concentration of cysteine in human blood plasma (Lopez, 2013). 50 mM concentration of cysteine is normally used to prepare CDM. A number of genes/gene clusters were differentially regulated under our tested conditions (**Table 3**). The expression of *spd-0447-49* and *spd-1098-99* was altered under our tested conditions. These genes belong to the glutamine regulon and their expression has been reported to be downregulated in the presence of a nitrogen source (Kloosterman et al., 2006b). The expression of important metal-related genes (*prtA*, *psaBC* and *spd-1402*) was downregulated under our tested conditions. These genes belong to the PsaR regulon and repressed by transcriptional regulator PsaR in the presence of manganese (Johnston et al., 2006). These genes have been shown to have role in virulence of pneumococcus (Kloosterman et al., 2008). Therefore, it might be interesting to further explore the role of cysteine in the regulation of these genes.

**Table 3:** Summary of transcriptome comparison of *S. pneumoniae* D39 wild-type grown in CDM with 0.03 mM cysteine to grown in CDM with 50 mM cysteine.

D39 tag^a^	Function^b^	Ratio^c^
**Upregulated genes**		
*spd_0145*	Hypothetical protein	2.8
*spd_0146*	CAAX amino terminal protease family protein	2.2
*spd_0147*	CAAX amino terminal protease family protein	2.6
*spd_0148*	Transporter, major facilitator family protein	1.8
*spd_0150*	ABC transporter, substrate-binding protein	7.2
*spd_0151*	Lipoprotein, MetQ	4.5
*spd_0152*	Peptidase, M20/M25/M40 family protein	2.0
*spd_0153*	ABC transporter, ATP-binding protein	2.0
*spd_0154*	ABC transporter, permease protein, putative	1.6
*spd_0373*	Hypothetical protein	3.2
*spd_0431*	Hypothetical protein	1.5
*spd_0510*	5-methyltetrahydropteroyltriglutamate–homocysteine *S*-methyltransferase, MetE	8.3
*spd_0511*	5,10-methylenetetrahydrofolate reductase, MetF	7.9
*spd_0540*	Amino acid ABC transporter, amino acid-binding protein, putative, GshT	4.8
*spd_0615*	ABC transporter substrate-binding protein, authentic truncation	3.2
*spd_0616*	Amino acid ABC transporter, ATP-binding protein	1.9
*spd_0617*	Amino acid ABC transporter, permease protein	2.0
*spd_0618*	Amino acid ABC transporter, permease protein	1.6
*spd_1073*	*O*-acetylhomoserine aminocarboxypropyltransferase/cysteine synthase	1.6
*spd_1074*	Hypothetical protein	1.5
*spd_1290*	Cystine ABC transporter, permease protein, TcyB	1.5
*spd_1352*	Aminotransferase, class II, Csd	2.3
*spd_1353*	Cys/Met metabolism PLP-dependent enzyme, putative, MetB	1.6
*spd_1406*	Homoserine *O*-succinyltransferase, MetA	1.7
*spd_1898*	Hypothetical protein	4.5
*spd_1899*	Glutamine amidotransferase, class 1, YvdE	6.9
*spd_2037*	Cysteine synthase A, CysK	3.7
**Downregulated genes**		
*spd_0447*	Transcriptional regulator, GlnR	-2.1
*spd_0448*	Glutamine synthetase, GlnA	-2.7
*spd_0449*	Hypothetical protein	-1.5
*spd_0558*	Cell wall-associated serine protease, PrtA	-4.4
*spd_1098*	Amino acid ABC transporter, GlnP	-1.9
*spd_1099*	Amino acid ABC transporter, GlnQ	-2.0
*spd_1402*	Non-heme iron-containing ferritin	-2.6
*spd_1461*	Manganese ABC transporter, ATP-binding protein, PsaB	-4.0
*spd_1462*	Manganese ABC transporter, permease protein, putative, PsaC	-4.0
*spd_1463*	ABC transporter, substrate binding lipoprotein	-4.0

*^a^Gene numbers refer to D39 locus tags. ^b^D39 annotation/TIGR4 annotation (Lanie et al., 2007), ^c^Ratio represents the fold increase/decrease in the expression of genes in CDM with 0.03 mM cysteine to CDM with 50 mM cysteine. Errors in the ratios never exceeded 10% of the given values*.

The expression of putative methionine/cysteine transport and biosynthesis pathway genes (*spd-0150*, *metQ*, *spd-0431*, *metEF*, *gshT*, *spd-0618*, *fhs, tcyB*, *metB*-*csd*, *metA*, *spd-1898*, *yvdE*, and *cysK*) was significantly upregulated in the presence of 0.03 mM cysteine. The role of methionine in regulation of these genes has been demonstrated in our recent study (Afzal et al., 2016). Furthermore, we showed that a transcriptional regulator CmhR acts as a transcriptional activator of *fhs*, *folD*, *metB*, *metEF*, *metQ*, and *spd-0431*. However, regulatory mechanism of *spd-0150*, *gshT*, *spd-0618*, *tcyB*, *metA*, *spd-1898*, *yvdE*, and *cysK* is not explored. Therefore, we decided to further explore the role of cysteine in the regulation of these genes.

### Confirmation of Cysteine-Dependent Expression of *spd-0150*, *metQ*, *spd-0431*, *metEF*, *gshT*, *spd-0618*, *fhs, tcyB*, *metB*-*csd*, *metA*, *spd-1898*, *yvdE*, and *cysK*

**FIGURE 1:**
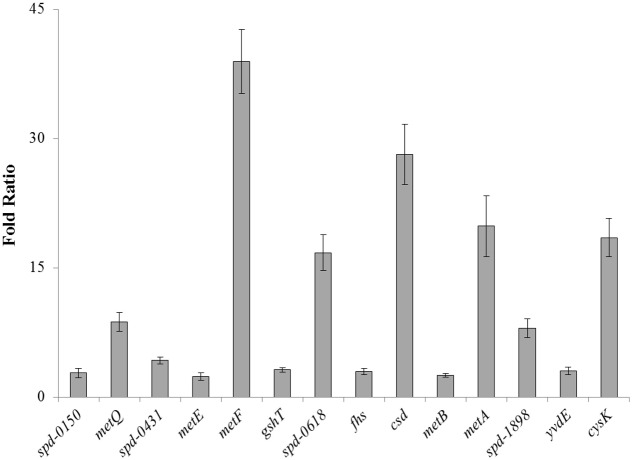
**The relative increase in the expression of *spd-0150*, *metQ*, *spd-0431*, *metEF*, *gshT*, *spd-0618*, *fhs, tcyB*, *metB*-*csd*, *metA*, *spd-1898*, *yvdE*, and *cysK* in *S. pneumoniae* D39 wild-type grown in CDM with 0.03 mM cysteine compared to that grown in CDM with 50 mM cysteine.** The expression of these genes was normalized with housekeeping gene *gyrA*.

To confirm our microarray results and to study the expression of *spd-0150*, *metQ*, *spd-0431*, *metEF*, *gshT*, *spd-0618*, *fhs, tcyB*, *metB*-*csd*, *metA*, *spd-1898*, *yvdE*, and *cysK* under limiting cysteine concentration in CDM, we performed quantitative RT-PCR on these genes. Our quantitative RT-PCR results demonstrated that the expression of these genes was increased significantly in CDM with 0.03 mM cysteine, when compared to 50 mM (**Figure 1**). These data not only confirms our microarray results mentioned above, but also suggests a direct role of these genes in cysteine transport and biosynthesis.

There are three LysR-type transcriptional regulators in different bacteria, which have been shown to be involved in the regulation of sulfur amino acids (Sperandio et al., 2005, 2010). *S. pneumoniae* also has two LysR-type transcriptional regulators (CmhR and CmbR), which are proposed to be involved in the regulation of the sulfur amino acids (Novichkov et al., 2010). Our recent study has revealed the regulatory mechanism of CmhR in *S. pneumoniae* and demonstrates that CmhR acts as a transcriptional activator of the *fhs*, *folD*, *metB*-*csd*, *metEF*, *metQ*, and *spd-0431* in the presence of methionine (Afzal et al., 2016). The presence of CmbR (putative Cysteine Methionine Biosynthesis Regulator) in *S. pneumoniae* suggests its involvement in the regulation of cysteine-responsive genes. Therefore, we decided to further study the role of transcriptional regulator CmbR in the regulation of cysteine transport and biosynthesis genes.

### Prediction of the CmbR Regulatory Site and the Role of CmbR as a Transcriptional Repressor of *spd-0150*, *metEF*, *gshT*, *spd-0618*, *tcyB*, *metA*, and *yvdE*

**FIGURE 2:**
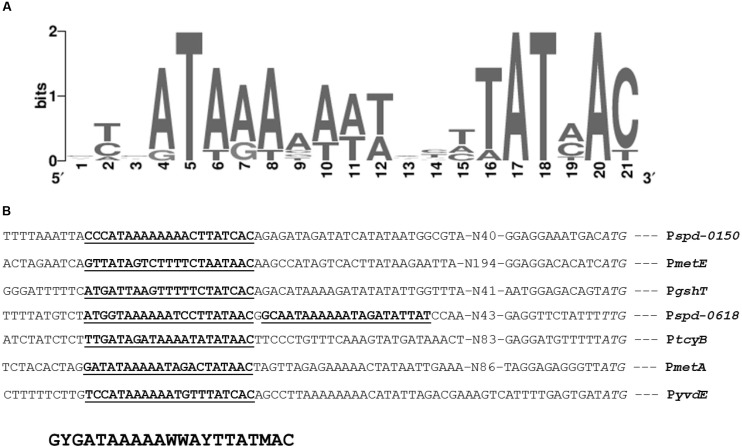
**Identification of the CmbR operator site. (A)** Weight matrix of the identified CmbR operator site in the promoter region of *spd-0150*, *metEF*, *gshT*, *spd-0618*, *tcyB*, *metA*, and *yvdE*. **(B)** Position of the CmbR operator site in the promoter region of *spd-0150*, *metEF*, *gshT*, *spd-0618*, *tcyB*, *metA*, and *yvdE*. Translational start sites are italic and putative CmbR operator sites are bold and underlined.

**FIGURE 3:**
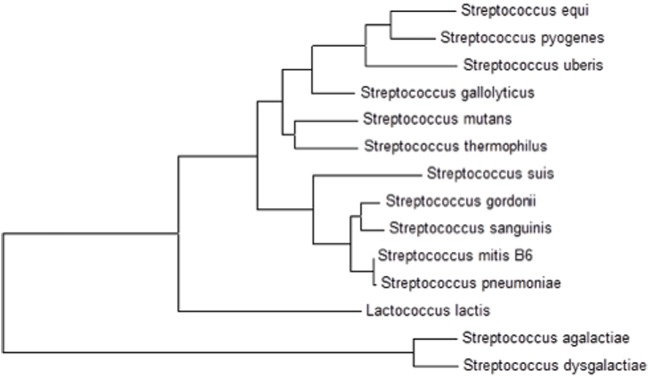
**A phylogenetic tree of CmbR in different streptococci showing conservation of CmbR in these streptococci**.

The presence of *cmbR* in the *S. pneumoniae* genome suggests its involvement in the regulation of cysteine-responsive genes. *cmbR* codes for the putative transcriptional regulator CmbR, which belongs to the LysR family of proteins. CmbR is a homolog of a LysR-type regulator (also called FhuR) of *Lactococcus lactis* and *Streptococcus mutans* (Fernández et al., 2002; Sperandio et al., 2005, 2010). To study the role of CmbR in *S. pneumoniae* D39, we analysed the promoter regions of cysteine-regulated genes and predicted a 21-bp palindromic-like sequence (that has high homology with the FhuR binding site of *L. lactis* and *S. mutans*) in the promoter regions of *spd-0150*, *metEF*, *gshT*, *spd-0618*, *tcyB*, *metA*, and *yvdE* indicating that CmbR regulon in *S. pneumoniae* D39 is comprised of these genes. This DNA sequence might serve as the CmbR operator site in *S. pneumoniae*. The CmbR site present in the promoter regions of *spd-0150*, *metEF*, *gshT*, *spd-0618*, *tcyB*, *metA*, and *yvdE* is shown in **Figure 2A**. A weight matrix of these CmbR sites (5′-GYGATAAAAAWWAYTTATMAC-3′) was constructed (**Figure 2B**). Promoter regions of these genes were also examined in other streptococcal species (*Streptococcus mitis*, *Streptococcus agalactiae*, *Streptococcus gallolyticus*, *Streptococcus gordonii*, *S. mutans*, *Streptococcus sanguinis*, *Streptococcus suis*, and *Streptococcus thermophilus*) to check whether the CmbR site is also conserved in those streptococci. The CmbR site is highly conserved in these streptococci as well. Moreover, we constructed a phylogenetic tree of CmbR present in different streptococci, which shows that it is highly conserved in these streptococci (**Figure 3**).

**FIGURE 4:**
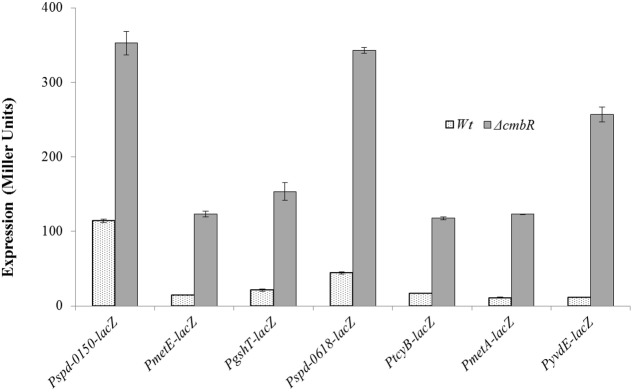
**Expression levels (in Miller units) of P*spd-0150-lacZ*, P*metE-lacZ*, P*gshT-lacZ*, P*spd-0618-lacZ*, P*tcyB-lacZ*, P*metA-lacZ*, and P*yvdE*-*lacZ* in CDM with 50 mM cysteine in *S. pneumoniae* D39 wild-type and D39 Δ*cmbR***.

To investigate the role of CmbR in the regulation of *spd-0150*, *metEF*, *gshT*, *spd-0618*, *tcyB*, *metA*, and *yvdE*, we performed β-galactosidase assays with the promoter *lacZ*-fusions that were constructed in the *S*. *pneumoniae* D39 wild-type and D39 *ΔcmhR* in CDM with 50 mM cysteine. We used CDM with 50 mM cysteine, because we assumed the role of CmbR as a transcriptional repressor of these genes in the presence of cysteine. The results of the β-galactosidase assays showed that the activity of P*spd-0150*-*lacZ*, P*metE*-*lacZ*, P*gshT*-*lacZ*, P*spd-0618*-*lacZ*, P*tcyB*-*lacZ*, P*metA*-*lacZ*, and P*yvdE*-*lacZ* increased significantly in D39 *ΔcmbR* compared to D39 wild-type in CDM with 50 mM cysteine (**Figure 4**). Increased expression of these promoters in D39 *ΔcmbR* indicates the role of CmbR as a transcriptional repressor of *spd-0150*, *metEF*, *gshT*, *spd-0618*, *tcyB*, *metA*, and *yvdE*.

### Confirmation of CmbR-Dependent Expression of *spd-0150*, *metEF*, *gshT*, *spd-0618*, *tcyB*, *metA*, and *yvdE*

**FIGURE 5:**
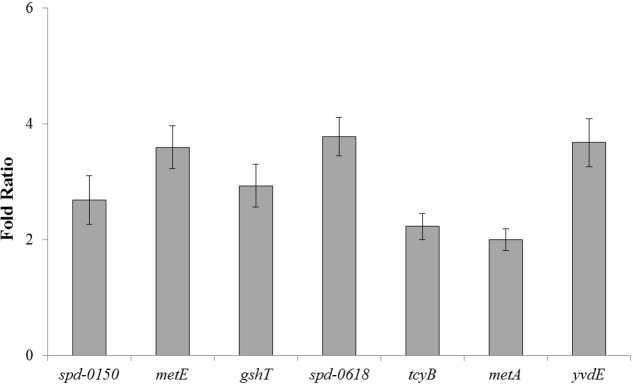
**The relative increase in the expression of *spd-0150*, *metEF*, *gshT*, *spd-0618*, *tcyB*, *metA*, and *yvdE* in *S. pneumoniae* D39 Δ*cmbR* compared to D39 wild-type grown in CDM with 50 mM cysteine.** The expression of these genes was normalized with that of housekeeping gene *gyrA*.

To further confirm the role of CmbR as a transcriptional repressor of *spd-0150*, *metEF*, *gshT*, *spd-0618*, *tcyB*, *metA*, and *yvdE*, we performed quantitative RT-PCR on these genes in the presence of 50mM cysteine. Our quantitative RT-PCR results demonstrated that the expression of *spd-0150*, *metEF*, *gshT*, *spd-0618*, *tcyB*, *metA*, and *yvdE* increased significantly in D39 *ΔcmbR* compared to the D39 wild-type (**Figure 5**). These data provide further confirmation of our results that CmbR acts as a transcriptional repressor of *spd-0150*, *metEF*, *gshT*, *spd-0618*, *tcyBC*, *metA*, and *yvdE*.

### Confirmation of a CmbR Operator Site in CmbR-Regulated Genes

**FIGURE 6:**
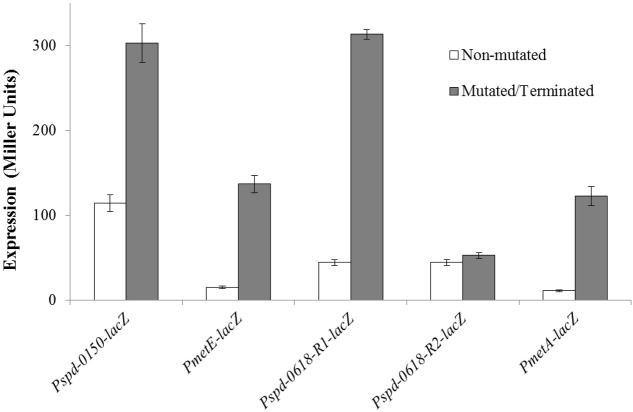
**Expression levels (in Miller units) of mutated/terminated and non-mutated CmbR sites in P*spd-0150-lacZ*, P*metEF-lacZ*, P*spd-0618-lacZ*, and P*metA-TER-lacZ* in *S. pneumoniae* D39 wild-type grown in CDM with 50 mM cysteine**.

To verify the functionality of the predicted CmbR operator site present in the promoter regions of the CmbR regulated genes (*spd-0150*, *metEF*, *gshT*, *spd-0618*, *tcyBC*, *metA*, and *yvdE*), we made transcriptional *lacZ***-**fusions of P*spd-0150*, P*metE*, P*spd-0618*, and P*metA*, where conserved bases in the *cmbR* sites were mutated in P*spd-0150* (5′-CCC**ATA**AAAAAAACTTATCAC-3′ to 5′-CCC**GCG**AAAAAAACTTATCAC-3′), P*metE* (5′-CTT**AT**AAGAATTACTA**AT**AAC-3′ to 5′-CTT**GC**AAGAATTACTA**GC**AAC-3′), P*spd-0618* (R1: 5′-ATGG**TA**AAAAATCCT**TAT**AAC-3′ to 5′-ATGG**CC**AAAAATCCT**GCG**AAC-3′ and R2: 5′-GCA**ATA**AAAAATAGA**TAT**TAT-3′ to 5′-GCA**GCG**AAAAATAGA**GCG**TAT-3′), and terminated in P*metA* (CmbR site is deleted), and β-galactosidase assays were performed on cells grown in CDM with 50 mM cysteine. β-galactosidase assays data revealed that mutating the CmbR operator site in P*spd-0150* and P*metE*, and deletion of the CmbR operator site in P*metA* led to significantly increased expression of these promoter *lacZ***-**fusions in the presence of 50 mM cysteine (**Figure 6**). These data confirm that the predicted CmbR sites present in the promoter regions of these genes are functional and intact in *S. pneumoniae* D39. Two putative operator sites of CmbR are present in P*spd-0618* (R1 and R2). We mutated both sites individually. We could only observe derepression (caused by CmbR) of P*spd-0618* when operator site 1 (R1) was mutated and did not witness any change in the activity of P*spd-0618* due to mutations in operator site 2 (R2). This suggests that only operator site 1 (R1) is a functional operator site for CmbR in P*spd-0618*.

## Discussion

The sulfur-containing amino acids cysteine and methionine play essential role in various metabolic processes in the cell, especially because of their sulfur group, which plays a vital role in the catalytic sites of many enzymes and participates in ion- and redox metabolism (Ayala-Castro et al., 2008). Involvement of multiple mechanisms in the regulation of these pathways in various groups of bacteria makes the regulatory phenomenon even more interesting, as it seems to evolve faster than that of many other regulatory pathways (Fernández et al., 2002; Sperandio et al., 2005, 2010). CmbR regulates most of the cysteine and methionine genes in *L. lactis*, where it binds to a 13-bp box centered 46–53 bp upstream of transcriptional start sites, with a second box with a same consensus sequence is located upstream of the first binding box (separated by 8–10 bp) (Fernández et al., 2002; Golic et al., 2005; Sperandio et al., 2005). In other members of the closely related Streptococcaceae family, the existence of a different motif upstream of several potential cysteine genes (Kovaleva and Gelfand, 2007) suggests that the regulation of sulfur amino acid metabolism may be diverse. In this study, we studied the cysteine-dependent gene expression and the role of CmbR in *S. pneumoniae* D39, and demonstrated that CmbR acts as a transcriptional repressor of *spd-0150*, *metEF*, *gshT*, *spd-0618*, *tcyB*, *metA*, and *yvdE*.

The number of transcriptional factors regulating cysteine/methionine genes varies among different bacteria. This number is three in *S. mutans* (MetR, CysR and HomR), where these transcriptional regulators are phylogenetically related (Sperandio et al., 2010). CysR activates the transcription of *cysK* (codes for the cysteine biosynthesis enzyme), *tcyABC*, *gshT* (code for the cysteine and glutathione transporter systems), and *homR*. HomR is needed for the activation of *metBC* (code for the methionine biosynthesis enzymes), *tcyDEFGH* (involved in cysteine transport) and thiosulfate metabolism genes. Control of HomR by CysR emulates a cascade regulation for sulfur amino acid metabolism in *S. mutans*. Similarly, MtaR has been found to have a role in the regulation of the cysteine/methionine metabolism in *S. agalactiae* (Shelver et al., 2003). MetJ and MetR, regulate the expression of methionine biosynthetic genes in *E. coli* and *S. enterica* serovar Typhimurium (Weissbach and Brot, 1991). The *E. coli met* genes (except for *metH*) are negatively regulated by MetJ, a transcriptional repressor, with SAM (S-adenosylmethionine) serving as a co-repressor (Saint-Girons et al., 1988). These genes are also under the positive influence of a LysR-type transcriptional regulator MetR, with homocysteine as a co-effector (Cai et al., 1989; Mares et al., 1992; Cowan et al., 1993). CmbR in *L. lactis* also activates most genes involved in the methionine and cysteine biosynthesis pathway in the absence of cysteine (Sperandio et al., 2005). The regulatory proteins mentioned above belong to LysR family of transcription factors, which is the most abundant family of regulators in bacteria (Maddocks and Oyston, 2008). These regulators control diverse biological pathways such as central metabolism, cell division, quorum sensing, virulence, motility, nitrogen fixation, oxidative stress responses, toxin production, attachment, and secretion. These transcriptional regulators act as either activators or repressors, and often are transcribed divergently with one of the regulated genes (Schell, 1993). LysR-family transcriptional regulators consist of two characteristic domains, an N-terminal HTH DNA binding domain (PF00126) and a C-terminal substrate binding domain (PF03466). There are two transcriptional regulators in *S. pneumoniae* that control the expression of the cysteine and methionine genes (CmhR and CmbR) (Novichkov et al., 2010). CmhR in *S. pneumoniae* belongs to the LysR family of transcriptional factors and has an HTH (helix-turn-helix) domain and a substrate binding domain of LysR-type transcriptional regulators (LTTRs). CmhR acts as a transcriptional activator of the *fhs*, *folD*, *metB*, *metEF*, *metQ*, and *spd-0431* genes and a 17-bp palindromic sequence (5′-TATAGTTTSAAACTATA-3′) is present in the promoter regions of CmhR-regulated genes in *S. pneumoniae* D39 (Afzal et al., 2016). There are two methionine transport systems [the methionine ABC uptake transporter (MUT)] (Merlin et al., 2002; Hullo et al., 2004) and a secondary transporter BcaP (den Hengst et al., 2006) in bacteria. *metD* codes for the MUT system in *E. coli* and also have the MetQ substrate binding protein (SBP), MetL trans-membrane permease and the MetN cytoplasmic ATP hydrolyzing protein (ATPase) (Merlin et al., 2002). The *spd-0150–54* locus codes for a methionine uptake ABC transporter in *S. pneumoniae* D39 and deletion of the gene encoding the lipoprotein MetQ resulted in a strain that had hampered growth in methionine-restricted medium and no measurable uptake of radioactive methionine (Basavanna et al., 2013). Furthermore, deletion of locus encoding MetEF (which is also part of the CmhR regulon) resulted in an increase in the growth defect of the *metQ* deletion strain in methionine-restricted medium and in blood plasma, reinforcing a role for the products of these genes in methionine synthesis (Basavanna et al., 2013). CmhR-regulated genes have important roles in the transport and biosynthesis of methionine. Csd and MetE are part of methionine synthesis pathway as Csd coverts cystathionine into homocysteine and MetE converts homocysteine into methionine (Kanehisa et al., 2014). Cystathionine and homocysteine can also be formed from homoserine, where *O*-acetyl-L-homoserine is converted into cystathionine by MetB. *O*-acetyl-L-homoserine can also be converted into homocysteine by MetB and SPD-1073-74 (*spd-1073-1074* encode an *O*-acetylhomoserine aminocarboxypropyltransferase/cysteine synthase and a hypothetical protein, respectively) (Kanehisa et al., 2014). Methionine can also be synthesized by other microbes as they may convert homoserine to homocysteine through addition of a sulfur group from either cysteine (involving MetABC), sulfide (involving MetA and CysD) or by using the SAM recycling pathway (MetK, Pfs and LuxS) (Kovaleva and Gelfand, 2007). MetE (methionine synthase) then methylates homocysteine in combination with MetF (methylenetetrahydrofolate reductase) and 5-methyltetrahydrofolate (FolD) provides it with the methyl group to form methionine (Ravanel et al., 1998; Kovaleva and Gelfand, 2007).

CmbR is the second one and acts as transcriptional repressor for *spd-0150*, *metEF*, *gshT*, *spd-0618*, *tcyB*, *metA*, and *yvdE*. The deletion of CmbR led to a significant increase in the activity of the CmbR-regulated promoters. Thus, it represents a different mode of regulation of the cysteine/methionine genes than in other related streptococci.

A number of metal-related genes are found to be differentially expressed in our tested conditions. These genes include *psaBC* and *prtA*, which belong to the PsaR regulon. *psaBCA* encode a Mn^2+^-dependent ABC transporter PsaBCA and *prtA* codes for a serine protease PrtA. The expression of these genes was also altered in our recent transcriptome of *S. pneumoniae* D39 grown in CDM with 0–10 mM methionine (Afzal et al., 2016). The DtxR-family transcriptional regulator PsaR represses the expression of the PsaR regulon in the presence of Mn^2+^ (Johnston et al., 2006). Zn^2+^, Ni^2+^ and Co^2+^ have been demonstrated to bind with PsaR and relieve the Mn^2+^-dependent repression of the PsaR regulon (Kloosterman et al., 2008; Manzoor et al., 2015a,b). The interplay or competition of metal ions plays a significant role in the regulation of metal-responsive genes. Competition of Mn^2+^ with Zn^2+^, Co^2+^, or Ni^2+^ in the regulation of the PsaR regulon by transcriptional regulator PsaR has already extensively been studied in *S. pneumoniae* (Kloosterman et al., 2008; Manzoor et al., 2015a,b). The significant changes in the expression of some of these metal-responsive genes in our study indicate the involvement of *S*-containing amino acids in this interplay as well, in an as yet unknown way.

## Author Contributions

MA planned the experiments, performed experiments and wrote the manuscript. SS planned the experiments and wrote the manuscript. IM performed the experiments. OK planned the experiments and wrote the manuscript.

## Conflict of Interest Statement

The authors declare that the research was conducted in the absence of any commercial or financial relationships that could be construed as a potential conflict of interest.
